# Supplementary material for: Activation and signaling mechanism revealed by GPR119-Gs complex structures
Source: Nat Commun. 2022 Nov 17;13:7033. doi: 10.1038/s41467-022-34696-6 (PMC9671963; doi:10.1038/s41467-022-34696-6)
Supplement: Supplementary file 1 — Supplementary Information [file 41467_2022_34696_MOESM1_ESM.pdf]

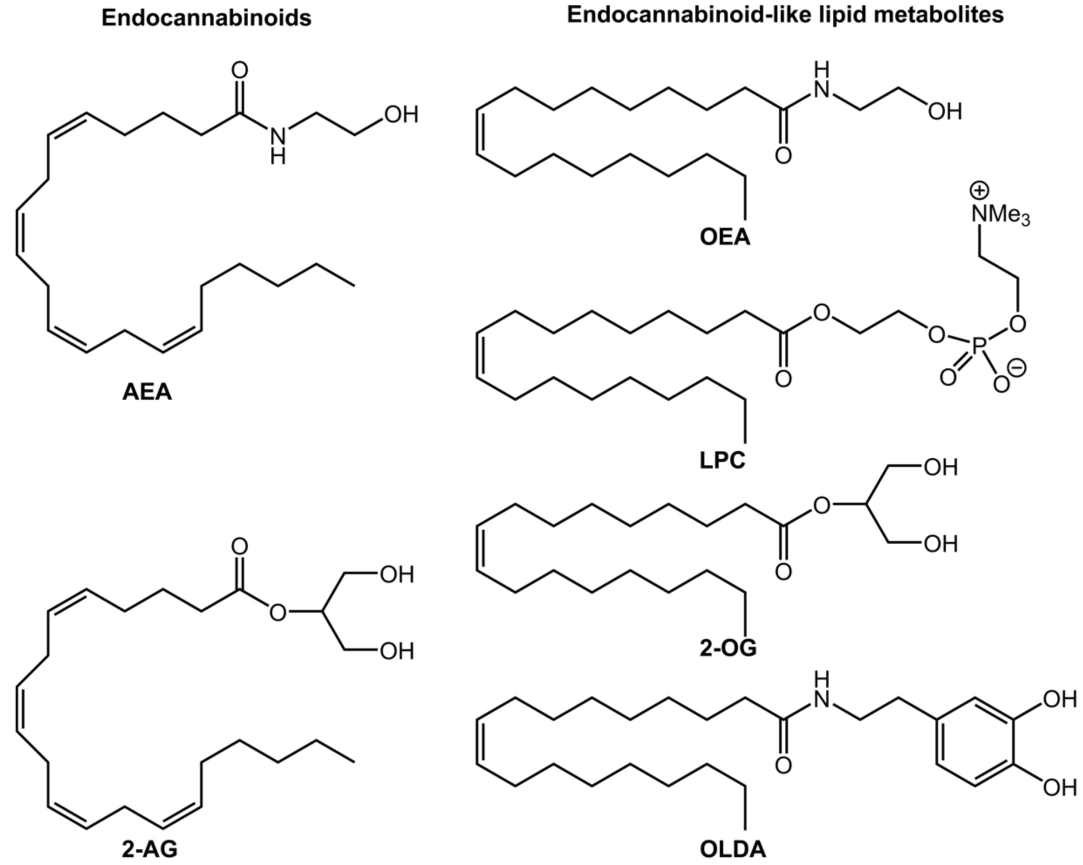

Supplementary Figure 1 | Chemical structures of endocannabinoids and endocannabinoid-like lipid metabolites. Anandamide (AEA) and 2-arachidonoyl glycerol (2-AG) are two canonical endocannabinoids. N-oleoylethanolamine (OEA), lysophosphatidylcholine (LPC), 2-oleoylglycerol (2-OG) and N-Oleoyldopamine (OLDA) are endocannabinoid-like lipid metabolites that activate GPR119.

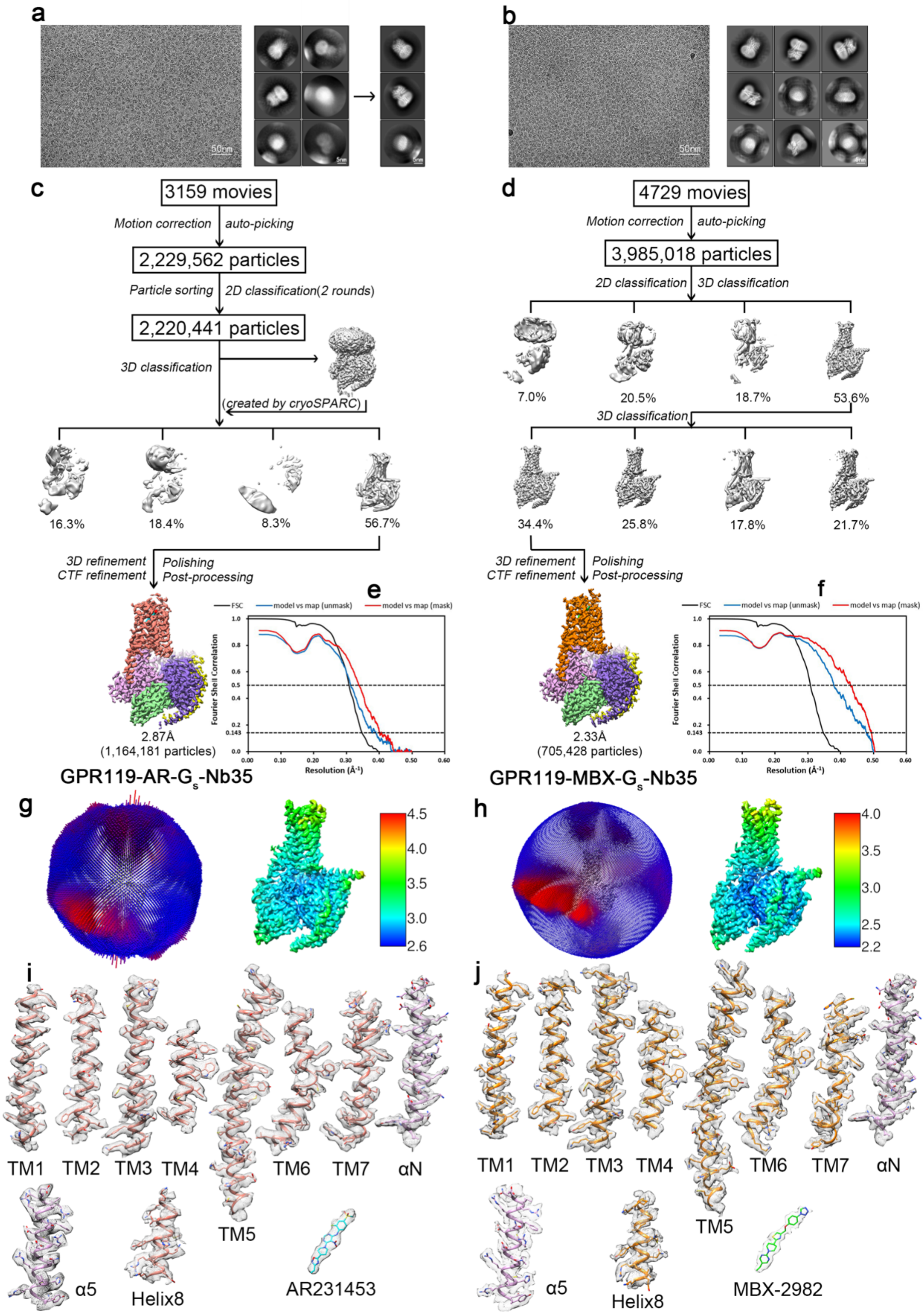

Supplementary Figure 2 | Cryo-EM reconstructions of AR231453-GPR119-G_s and MBX-2982-GPR119-G_s complexes. a, b, Representative motion-corrected micrographs and 2D class averages of AR231453-GPR119-G_s **(a)** and MBX-2982-

GPR119-G_s (**b**) complexes. A total of 3,159 and 4,729 micrographs were collected for AR231453-GPR119-G_s and MBX-2982-GPR119-G_s complex reconstruction respectively. **c, d,** Flow chart of EM data processing and 3D reconstruction of AR231453-GPR119-G_s (**c**) and MBX-2982-GPR119-G_s (**d**) complexes. **e, f,** Fourier shell correlation (FSC) curves for the final model of AR231453-GPR119-G_s (**e**) and MBX-2982-GPR119-G_s (**f**) complexes using the FSC=0.143 criterion indicate overall resolutions of 2.87 Å and 2.33 Å, respectively. **g, h,** Angle distribution maps and local resolution estimations of AR231453-GPR119-G_s (**g**) and MBX-2982-GPR119-G_s (**h**) complexes. **i, j,** Cryo-EM density maps and models of TM1-7, and Helix8 of GPR119, αN and α5 of $G\alpha_s$ and ligand of AR231453-GPR119-G_s (contoured at 0.016) (**i**) and MBX-2982-GPR119-G_s (contoured at 0.014) (**j**) complexes, respectively.

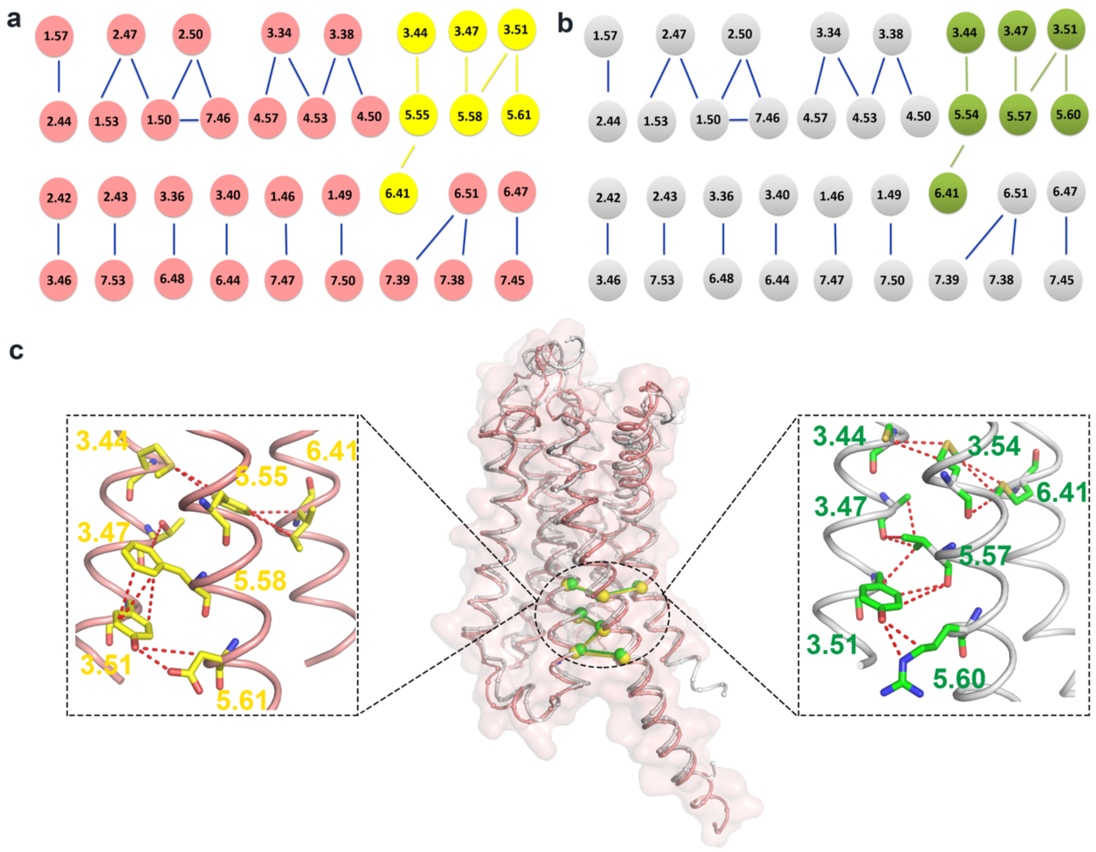

Supplementary Figure 3 | GPR119 displays a noncanonical consensus structural scaffold. a, b, Schematic representations of the consensus network of 24 inter-TM contacts mediated by 36 topologically equivalent amino acids of canonical class A GPCRs (Venkatakrishnan et al., 2013) (**a**) and GPR119 (**b**). The lines between a pair of circles indicate the presence of non-covalent contacts. Numbers denote Ballesteros–Weinstein numbering. **c,** Superimposition of GPR119 (pink, this study) and β2AR (gray, PDB ID: 3S6N, representative receptor of canonical class A GPCRs) showing five of the non-covalent contacts, all involving residues of TM5, are not conserved in GPR119. The Cαs of the 5 non-conserved inter-TM contacts shown as yellow (GPR119) and green (β2AR) spheres, linked by sticks, respectively. Insets show the detail interactions in GPR119 and β2AR.

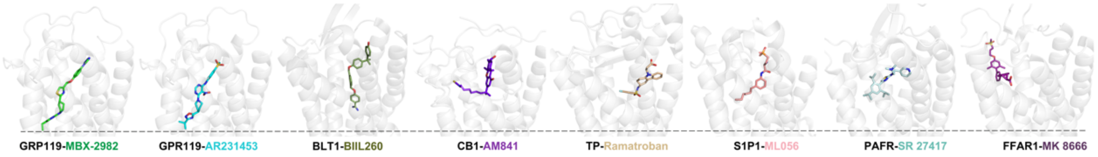

Supplementary Figure 4 | Comparison of the ligand-binding sites in different subtype lipid receptor structures. GPR119-MBX-2982 (this study), GPR119-AR231453 (this study), BLT1-BIIL260 (PDB ID: 5X33), CB1-AM841 (PDB ID: 6KPG), TP-Ramatroban (PDB ID: 6IIU), S1P1-ML056 (PDB ID: 3V2Y), PAFR-SR27417 (PDB ID: 5ZKP), FFAR1-MK 8666 (PDB ID: 5TZR) are superimposed and shown in grey cartoon, with the ligands shown in green, cyan, grass green, dark purple, wheat, pink, light blue and purple sticks, respectively. The black dashed line shows the deepest binding site that MBX-2982 binding in the GPR119 transmembrane helical bundle.

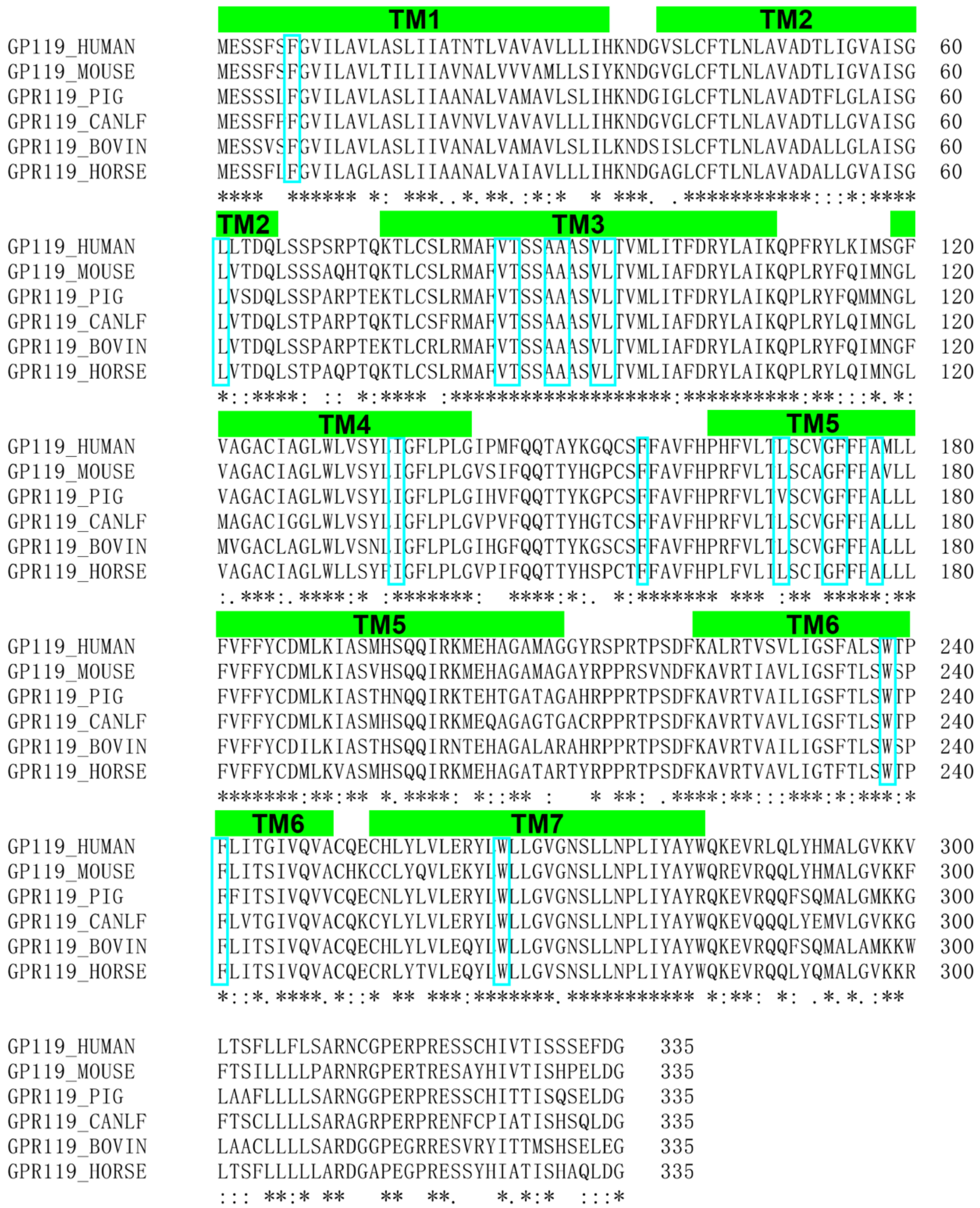

Supplementary figure 5 | Sequence alignment of GPR119 across the vertebrate lineage. The cyan boxes denote positions that make contact with AR231453 or MBX-2982 within a 4 Å cut-off. The alignment was generated using Clustal Omega (http://www.ebi.ac.uk/Tool/msa/clustalo/).

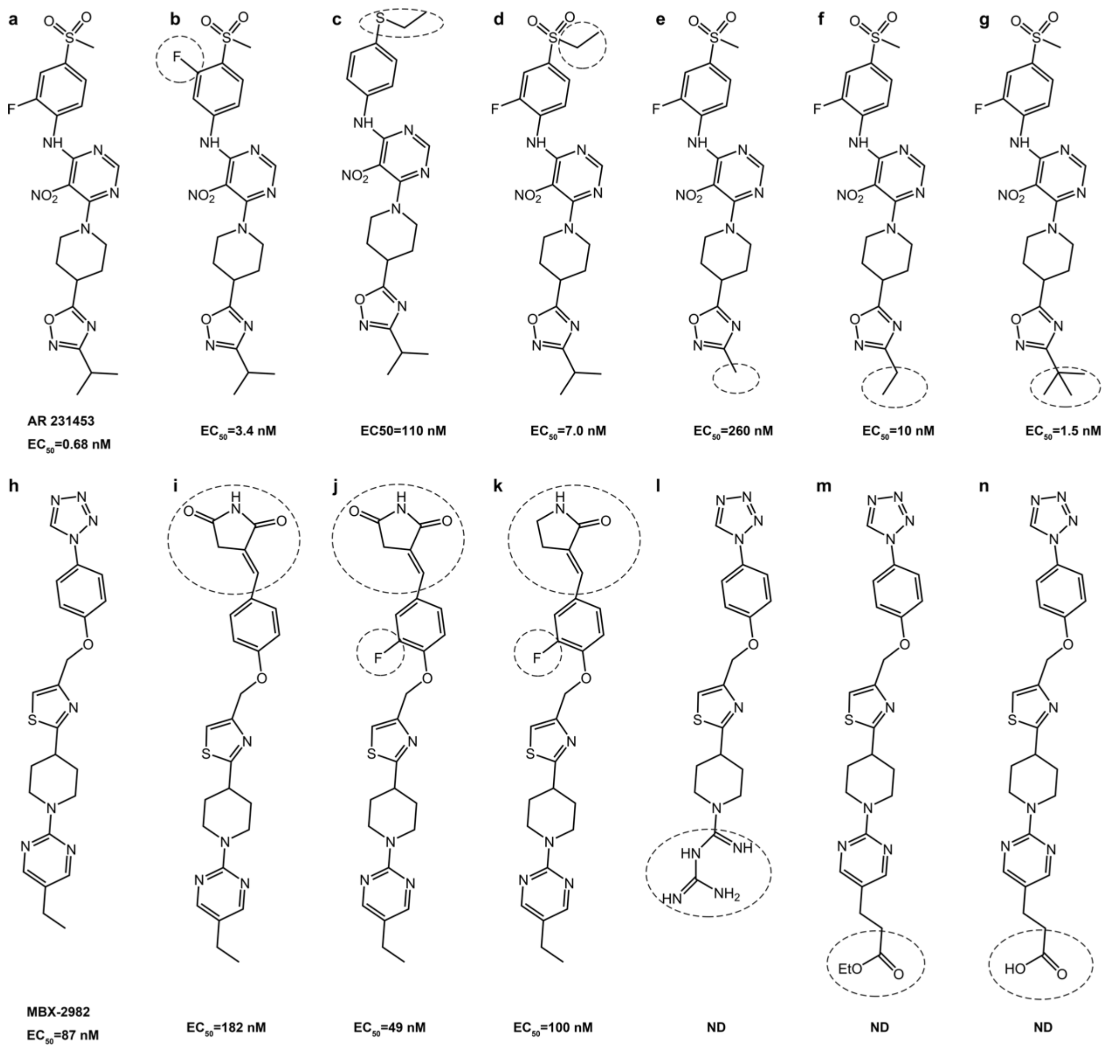

Supplementary figure 6 | Chemical structures of different derivatives of AR231453 and MBX-2982. (a-n) Different moieties are indicated by dashed ovals. The EC50 shown below the chemical structures are from previous studies (Kim et al., 2017; Semple et al., 2008).

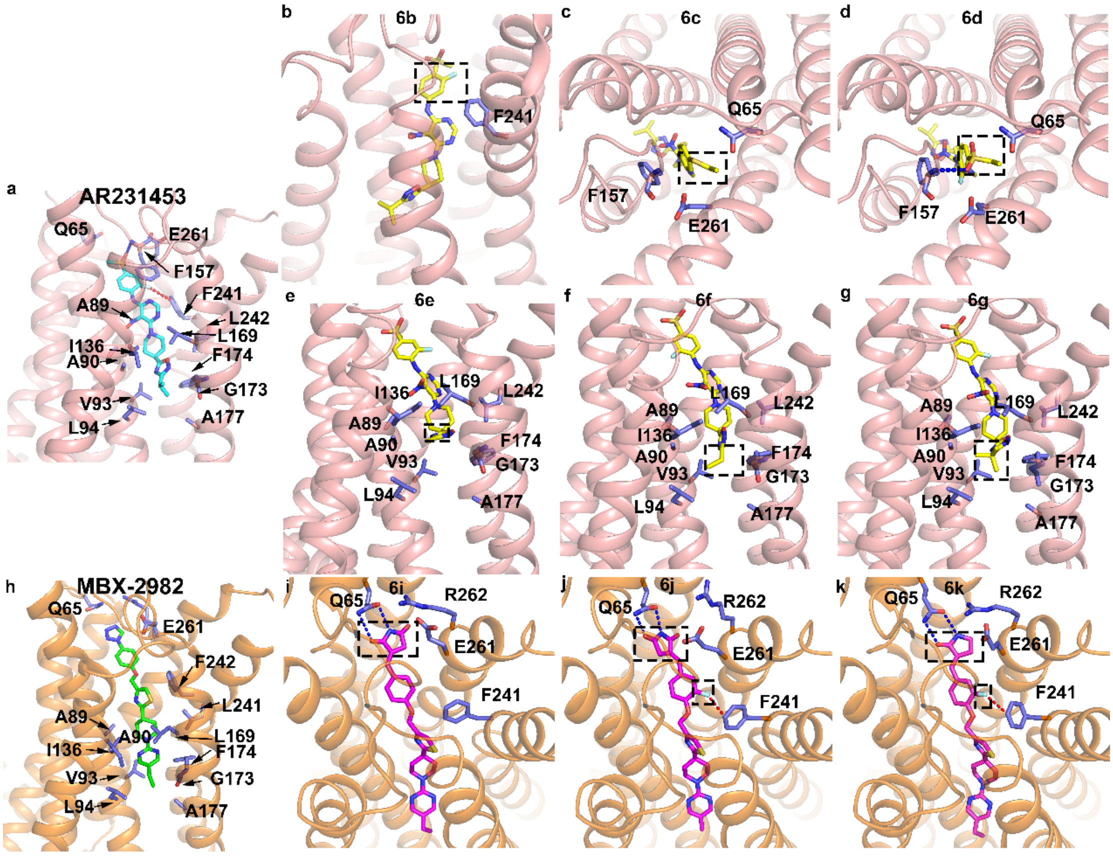

Supplementary figure 7 | Docking model of different derivatives of AR231453 and MBX-2982. (a-k) Representative binding mode of each ligand is shown. Different moieties among these ligands are highlighted by black dashed rectangles. The halogen…π interactions and hydrogen bonds are displayed by red and blue dashed lines, respectively.

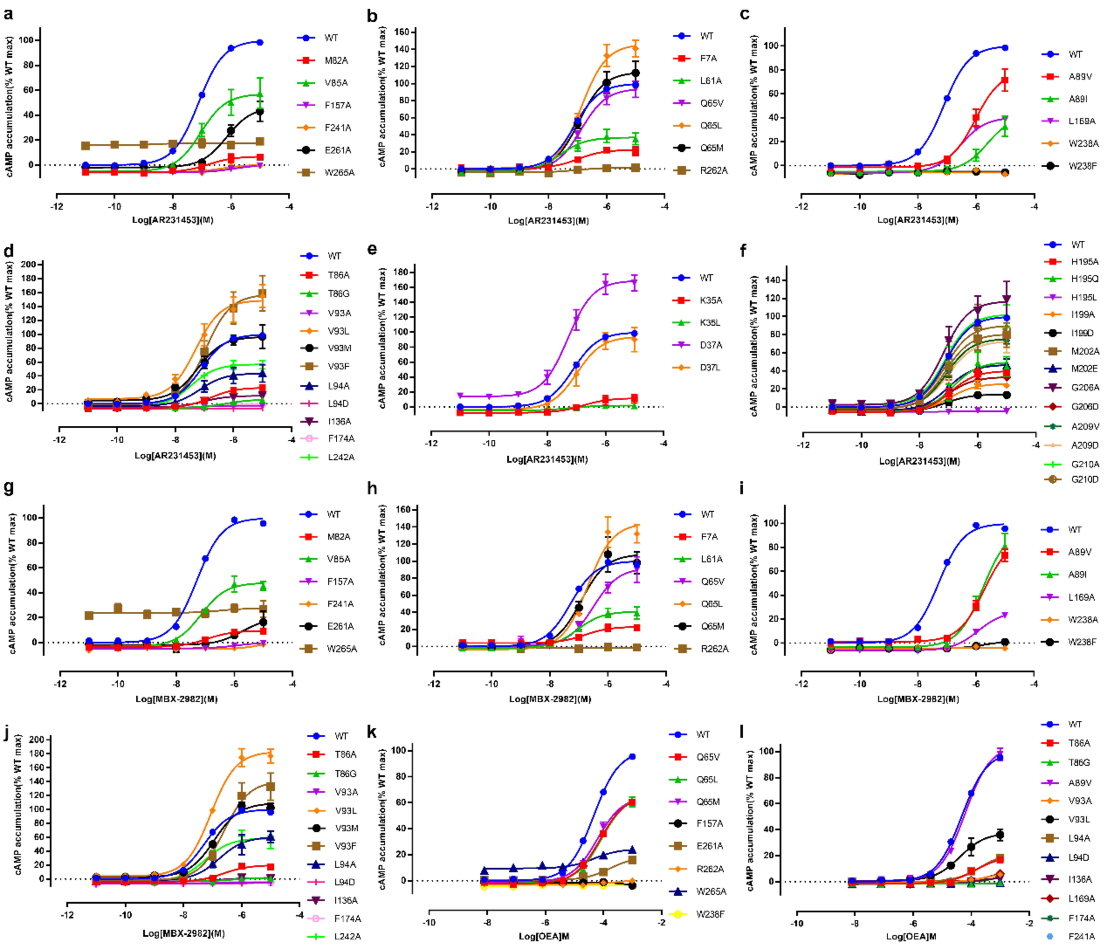

Supplementary figure 8 | Signaling assay of GPR119. Data are shown as mean ± s.e.m. (bars) from three independent experiments performed in technical triplicate. **a, b, c, d, e, f,** AR231453-induced cAMP accumulation assay. The detailed statistical evaluation and expression level are provided by Supplementary Table 3. **g, h, i, j,** MBX-2982-induced cAMP accumulation assay. The detailed statistical evaluation and expression level are provided by Supplementary Table 3. **k, l,** OEA-induced cAMP accumulation assay. The detailed statistical evaluation and expression level are provided by Supplementary Table 4.

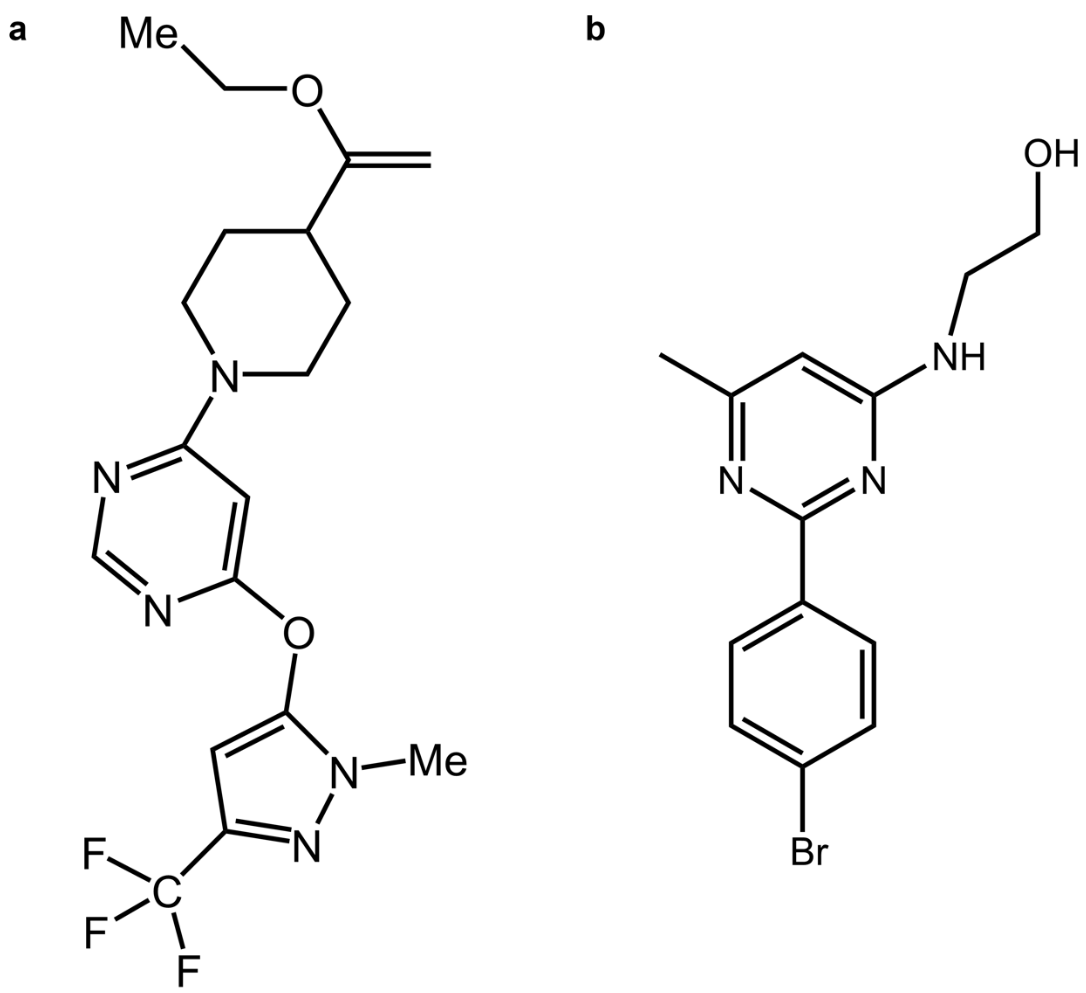

Supplementary figure 9 | Chemical structures of additional GPR119 ligands. a, An inverse agonist relative to AR231453. **b**, agonist AS1269574.

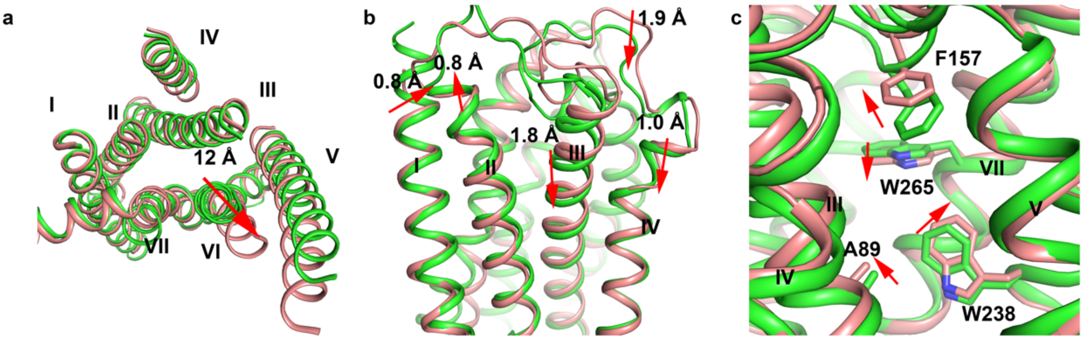

Supplementary figure 10 | Structural comparison between agonist-bound and the predicted ligand-free GPR119 structures. a, Superimposition of the AR231453-bound (light pink color) and the predicted ligand-free (green color) GPR119 structures reveals a 12 Å outward movement of TM6 when measured at the Cα carbon of Asp220. **b,** The overall conformational changes closed to the ligand binding site. **c,** The ligand-binding site in the predicted ligand-free GPR119 structure is distorted.

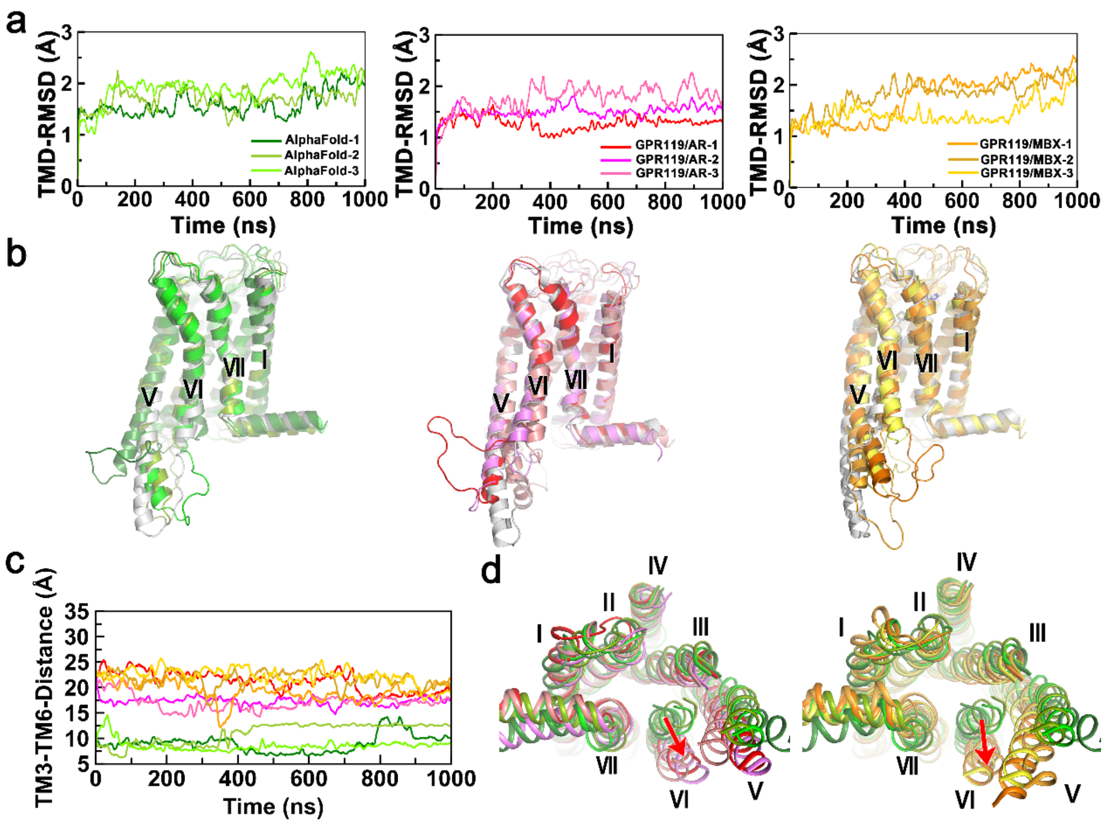

Supplementary figure 11 | GPR119 conformations in MD simulations. a, Time dependences of r.m.s.d values of the transmembrane domain in three simulation systems. The values were calculated using the Cα atoms of S6-L32(TM1), S40-D64 (TM2), K75-I107(TM3), V121-L141 (TM4), H164-I199 (TM5), A223-A250 (TM6) and L258-W282 (TM7). **b,** Superimposition of the representative structures in each simulation and the initial protein conformation for each system. The agonist-bound and the predicted ligand-free GPR119 structures were shown in white. The representative structures were generated from conformation clustering based on the r.m.s.d values of the last 200 ns shown in (a) and were colored according to (a). **c,** The TM3-TM6 distances during each simulation. Values were generated by calculating the Cα – Cα distance between A106 and D220. **d,** Structural comparison between two agonist-

bound systems and the ligand-free system from the intracellular view. The snapshots were the same as those in (b).

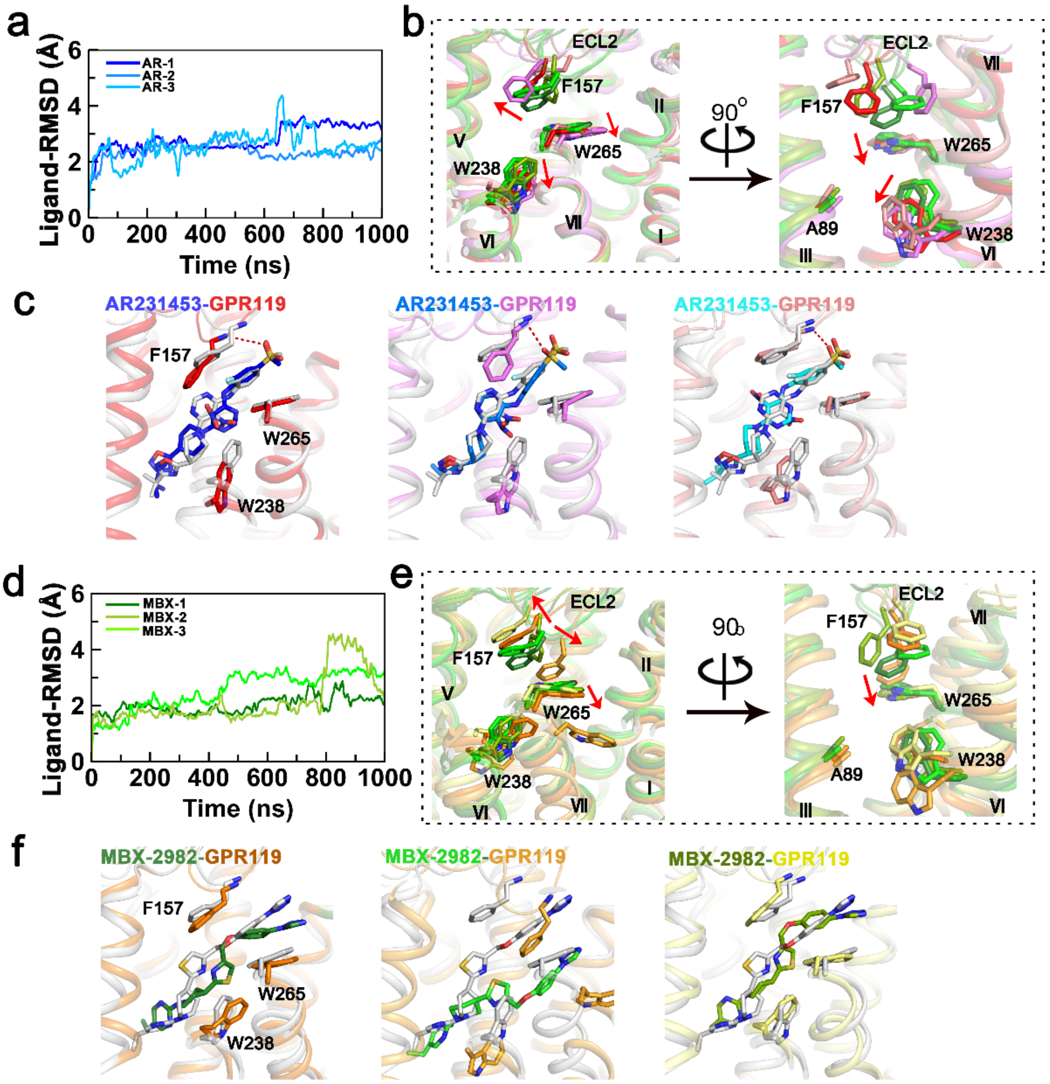

Supplementary figure 12 | Agonist binding in MD simulations. a, The r.m.s.d values of AR231453 in the simulation. **b,** Conformational comparison of important residues around the ligand-binding pocket in AR231453-bound system and ligand-free system with the same color as Supplementary figure 10a. **c,** Representative binding pose of AR231453 in each simulation. **d,** The r.m.s.d values of MBX-2982 in the simulation. **e,** Conformational comparison of important residues in MBX-2982-bound system and

ligand-free system. **f,** Representative binding pose of MBX-2982 in each simulation. The typical structures shown were obtained from conformation clustering using the ligand r.m.s.d values in the last 200 ns trajectories. Proteins were colored according to Supplementary figure 10a and ligand were shown in the same color scheme as in (a). Hydrogen bonds were represented by red dash lines.

Supplementary Table 1 | Cryo-EM data collection, model refinement and validation statistics

	GPR119-AR-Gs-Nb35 (PDB : 7WCN) (EMDB : 32425)	GPR119-MBX-Gs-Nb35 (PDB : 7WCM) (EMDB : 32424)
**Data collection and processing**		
Magnification	105,000	105,000
Voltage (kV)	300	300
Detector	Gatan K3	Gatan K3
Electron exposure ($e^-/Å^2$)	54 (40 frames)	54 (40 frames)
Defocus range (μm)	-1.0 ~ -1.5	-1.0 ~ -1.5
Pixel size (Å)	0.851	0.851
Symmetry imposed	C1	C1
Initial particle projections (no.)	2,229,562	3,985,018
Final particle projections (no.)	1,164,181	705,428
Map resolution (Å)	2.87	2.33
FSC threshold	0.143	0.143
Map resolution range (Å)	2.77–6.15	2.23-6.45
**Refinement**		
Initial model used	6LMK	GPR119-AR-Gs-Nb35
Refinement package	Phenix.real_space_refine	Phenix.real_space_refine
Model resolution (Å)	2.95	2.37
FSC threshold	0.5	0.5
Map sharpening B-factor ($Å^2$)	-83	-36
Model composition		
Non-hydrogen atoms	8252	8232
Protein residues	1050	1047
Ligand	1	1
R.m.s. deviations		
Bond lengths (Å)	0.004	0.002
Bond angles (°)	0.618	0.562
Validation		
MolProbity score	1.48	1.26
Clashscore	4.32	3.66
Rotamer outliers (%)	0.00	0.00
CaBLAM outliers (%)	2.31	1.38
Ramachandran plot		
Favored (%)	96.13	97.48
Allowed (%)	3.87	2.52
Disallowed (%)	0.00	0.00

Supplementary Table 2 | AR231453 or MBX-2982-induced cAMP accumulation assays of GPR119.

Mutants	AR231453						MBX-2982						Expression[e]	
	EC_{50}[a] (nM)	pEC_{50}		Span[c]		n[d]	EC_{50}[a] (nM)	pEC_{50}		Span[c]		n[d]		
		mean ±s.e.m. [b]	*P* value	% of WT [b]	*P* value			mean ±s.e.m. [b]	*P* value	% of WT [b]	*P* value		% of WT [b]	*P* value
Wild type	76.9	7.11±0.02		100±1		18	51.5	7.29±0.03		100±1		18	100	
Construct 1[f]	55.3	7.26±0.04	0.9728	96±2	0.9915	3	131.5	6.88±0.04	0.1171	82±2	0.0182	3	137±6*	0.0012
F7$^{1.35}$A	104.0	6.98±0.26	0.9950	22±3***	＜0.0001	3	161.9	6.79±0.21	0.0326	19±2***	＜0.0001	3	53±6***	＜0.0001
K35^{ICL1}A	169.2	6.77±0.36	0.2520	20±3***	＜0.0001	3	/						67±13*	0.0062
K35^{ICL1}L	ND	ND	ND	ND	ND	3	/						64±5*	0.0018
D37^{ICL1}A	50.8	7.29±0.11	0.9896	155±7***	＜0.0001	3	/						102±4	0.9997
D37^{ICL1}L	104.5	6.98±0.18	0.9073	98±8	0.9994	3	/						72±5	0.0373
L61$^{2.60}$A	25.6	7.59±0.23	0.0493	40±4***	＜0.0001	3	87.7	7.06±0.16	0.7692	43±3***	＜0.0001	3	59±6*	0.0002
Q65$^{2.64}$V	132.0	6.88±0.09	0.7871	96±4	0.9958	3	313.8	6.5±0.17***	＜0.0001	91±7	0.6103	3	83±5	0.5840
Q65$^{2.64}$L	133.0	6.88±0.08	0.7871	148±6***	＜0.0001	3	200.3	6.7±0.12*	0.0057	148±9***	＜0.0001	3	95±3	0.9992
Q65$^{2.64}$M	112.8	6.95±0.12	0.9794	115±6	0.0918	3	119.5	6.92±0.17	0.2000	109±9	0.6103	3	127±7	0.0516

M82$^{3.29}$A	ND	ND	ND	ND	ND	3	ND	ND	ND	ND	ND	3	90±7	0.9980
V85$^{3.32}$A	91.2	7.04±0.19	0.9994	62±5***	＜0.0001	3	71.9	7.14±0.11	0.9870	51±3***	＜0.0001	3	80±9	0.3321
T86$^{3.33}$A	160.7	6.79±0.14	0.3867	29±2***	＜0.0001	3	232.5	6.63±0.13*	0.0015	24±1***	＜0.0001	3	65±6*	0.0028
T86$^{3.33}$G	ND	ND	ND	ND	ND	3	ND	ND	ND	ND	ND	3	51±9***	＜0.0001
A89$^{3.36}$V	951.4	6.02±0.13***	＜0.0001	80±6*	0.0087	3	1879.0	5.73±0.08***	＜0.0001	86±4	0.1174	3	114±11	0.8532
A89$^{3.36}$I	2446.0	5.61±0.26***	＜0.0001	47±8***	＜0.0001	3	1759.0	5.76±0.11***	＜0.0001	100±7	0.9999	3	104±11	0.9994
A89$^{3.36}$L	2030.0	5.69±0.24***	＜0.0001	37±6***	＜0.0001	3	92.7	7.03±0.14	0.6269	108±7	0.7502	3	81±5	0.4077
V93$^{3.40}$A	ND	ND	ND	ND	ND	3	ND	ND	ND	ND	ND	3	79±3	0.2659
V93$^{3.40}$L	49.6	7.31±0.15	0.8976	142±9***	＜0.0001	3	87.6	7.06±0.07	0.7692	179±6***	＜0.0001	3	104±5	0.9994
V93$^{3.40}$F	109.2	6.96±0.16	0.9877	158±12***	＜0.0001	3	251.3	6.6±0.15*	0.0008	143±10***	＜0.0001	3	60±6*	0.0003
V93$^{3.40}$M	57.8	7.24±0.16	0.9950	92±7	0.7714	3	115.7	6.94±0.07	0.2559	106±4	0.9526	3	108±3	0.9986
L94$^{3.41}$A	69.2	7.16±0.29	0.9996	47±6***	＜0.0001	3	170.0	6.77±0.2	0.0206	63±6***	＜0.0001	3	92±7	0.9986
L94$^{3.41}$D	ND	ND	ND	ND	ND	3	ND	ND	ND	ND	ND	3	58±6*	0.0001

I136$^{4.56}$A	ND	ND	ND	ND	ND	3	ND	ND	ND	ND	ND	3	80±14	0.3321
F157^{ECL2}A	ND	ND	ND	ND	ND	3	ND	ND	ND	ND	ND	3	91±31	0.9983
L169$^{5.43}$A	289.7	6.54±0.04	0.0112	46±1***	＜0.0001	3	1179.0	5.93±0.08***	＜0.0001	33±2***	＜0.0001	3	78±16	0.2099
F174$^{5.48}$A	ND	ND	ND	ND	ND	3	ND	ND	ND	ND	ND	3	91±7	0.9983
H195$^{5.69}$A	117.7	6.93±0.13	0.9073	46±3***	＜0.0001	3	/						81±4	0.4077
H195$^{5.69}$Q	136.1	6.87±0.25	0.6616	55±6***	＜0.0001	3	/						97±3	0.9996
H195$^{5.69}$L	ND	ND	ND	ND	ND	3	/						92±4	0.9986
I199$^{5.73}$A	110.8	6.96±0.15	0.9715	30±2***	＜0.0001	3	/						125±7	0.0944
I199$^{5.73}$D	ND	ND	ND	ND	ND	3	/						106±5	0.9990
M202$^{5.76}$E	94.0	7.03±0.12	0.9993	51±3***	＜0.0001	3	/						106±6	0.9990
M202$^{5.76}$A	76.2	7.12±0.17	0.9999	83±7	0.0204	3	/						95±6	0.9992
G206$^{5.80}$A	66.0	7.18±0.18	0.9994	114±9	0.0836	3	/						85±4	0.7707
G206$^{5.80}$D	100.0	7±0.11	0.9958	36±2***	＜0.0001	3	/						81±5	0.4077
A209$^{5.83}$V	84.0	7.08±0.1	0.9997	77±4*	0.0007	3	/						94±7	0.9990
A209$^{5.83}$D	95.8	7.02±0.15	0.9991	73±5***	＜0.0001	3	/						87±7	0.9187

G210$^{5.84}$A	76.6	7.12±0.13	0.9999	100±6	0.9999	3	/						68±6*	0.0091
G210$^{5.84}$D	76.0	7.12±0.19	0.9999	90±8	0.3753	3	/						65±6*	0.0028
W238$^{6.48}$F	ND	ND	ND	ND	ND	3	ND	ND	ND	ND	ND	3	46±2***	＜0.0001
W238$^{6.48}$A	ND	ND	ND	ND	ND	3	ND	ND	ND	ND	ND	3	39±4***	＜0.0001
F241$^{6.51}$A	ND	ND	ND	ND	ND	3	ND	ND	ND	ND	ND	3	61±14*	0.0005
L242$^{6.52}$A	34.8	7.46±0.12	0.2810	61±3***	＜0.0001	3	56.6	7.25±0.16	0.9997	62±4***	＜0.0001	3	100±8	0.9999
E261$^{7.35}$A	707.1	6.15±0.16***	＜0.0001	49±4***	＜0.0001	3	ND	ND	ND	ND	ND	3	87±5	0.9187
R262$^{7.36}$A	ND	ND	ND	ND	ND	3	ND	ND	ND	ND	ND	3	110±9	0.9980
W265$^{7.39}$A	ND	ND	ND	ND	ND	3	ND	ND	ND	ND	ND	3	63±10*	0.0012

aEC_{50} values were determined after 0.5 h stimulation by increasing concentrations of AR231453 or MBX-2982 at room temperature.

bData are shown as mean ± s.e.m. from at least three independent experiments performed in triplicate.**P*<0.01; ****P*<0.0001 by one-way ANOVA followed by Dunnett's post-test, compared with the response of the WT.

cThe span is defined as the window between the maximal AR231453 or MBX-2982 response (E_{max}) and the vehicle (no ligand). ND (not determined) refers to data where a robust concentration response curve could not be established within the concentration range tested, such that an E_{max} was not reached and therefore span could not be calculated.

dProtein expression levels of GPR119 constructs at the cell surface were determined in parallel by flowcytometry with an anti-FLAG antibody and reported as percent compared to the WT GPR119 from at least three independent measurements.

eProtein expression levels of GPR119 constructs at the cell surface were determined in parallel by flowcytometry with an anti-FLAG antibody and reported as percent compared to the WT GPR119 from at least three independent measurements.

[f]Construct 1 means bRIL-GPR119 with a single mutation S237$^{6.47}$C.

Supplementary Table 3 | OEA-induced cAMP accumulation assays of GPR119.

Mutants	EC_{50}[a] (μM)	pEC_{50}		Span[c]		n[d]	Expression[e]	
		mean±s.e.m. [b]	*P* value	% of WT [b]	*P* value		% of WT [b]	*P* value
Wild type	46.84	4.33±0.02		100±1		6	100	
Q65$^{2.64}$V	76.98	4.11±0.04	0.2359	68±1***	<0.0001	3	83±5	0.8544
Q65$^{2.64}$L	86.92	4.06±0.05	0.1024	69±2***	<0.0001	3	95±3	>0.9999
Q65$^{2.64}$M	61.64	4.21±0.06	0.7849	66±2***	<0.0001	3	127±7	0.1721
T86$^{3.33}$A	144	3.84±0.13*	0.0013	21±2***	<0.0001	3	90±12	0.9994
T86$^{3.33}$G	ND	ND	ND	ND	ND	3	86±7	0.9702
A89$^{3.36}$V	61.93	4.21±0.04	0.7849	105±3	0.2580	3	81±4	0.7166
V93$^{3.40}$A	ND	ND	ND	ND	ND	3	76±7	0.3296
V93$^{3.40}$L	41.25	4.39±0.16	0.9910	37±3***	<0.0001	3	88±10	0.9941
L94$^{3.41}$A	198.4	3.70±0.14***	<0.0001	22±2***	<0.0001	3	75±8	0.2687
L94$^{3.41}$D	ND	ND	ND	ND	ND	3	38±2***	<0.0001
I136$^{4.56}$A	ND	ND	ND	ND	ND	3	58±5*	0.0025
F157^{ECL2}A	ND	ND	ND	ND	ND	3	73±10	0.1721
L169$^{5.43}$A	ND	ND	ND	ND	ND	3	77±5	0.3985
F174$^{5.48}$A	ND	ND	ND	ND	ND	3	73±8	0.1721
W238$^{6.48}$F	ND	ND	ND	ND	ND	3	87±23	0.9858
F241$^{6.51}$A	ND	ND	ND	ND	ND	3	64±4	0.0155
E261$^{7.35}$A	ND	ND	ND	ND	ND	3	70±2	0.0819
R262$^{7.36}$A	ND	ND	ND	ND	ND	3	110±9	0.9994
W265$^{7.39}$A	ND	ND	ND	ND	ND	3	67±9	0.0365

[a]EC_{50} values were determined after 0.5 h stimulation by increasing concentrations of AR231453 at room temperature.

[b]Data are shown as mean ± s.e.m. from at least three independent experiments performed in triplicate.**P*<0.01; ****P*<0.0001 by one-way ANOVA followed by Dunnett's post-test, compared with the response of the WT.

[c]The span is defined as the window between the maximal AR231453 response (E_{max}) and the vehicle (no OEA). ND (not determined) refers to data where a robust concentration response curve could not be established within the concentration range tested, such that an E_{max} was not reached and therefore span could not be calculated.
[d]Sample size; the number of independent experiments performed in triplicate.
[e]Protein expression levels of GPR119 constructs at the cell surface were determined in parallel by flowcytometry with an anti-FLAG antibody and reported as percent compared to the WT GPR119 from at least three independent measurements.
