# Supplementary material for: Activation and signaling mechanism revealed by GPR119-Gs complex structures
Source: Nat Commun. 2022 Nov 17;13:7033. doi: 10.1038/s41467-022-34696-6 (PMC9671963; doi:10.1038/s41467-022-34696-6)
Supplement: Supplementary file 3 — Description of Additional Supplementary Files [file 41467_2022_34696_MOESM3_ESM.pdf]

File Name: Supplementary Data 1
Description: Consensus network of inter-TM contacts in active/inactive states in canonical class A GPCRs
