# Supplementary material for: Activation and signaling mechanism revealed by GPR119-Gs complex structures
Source: Nat Commun. 2022 Nov 17;13:7033. doi: 10.1038/s41467-022-34696-6 (PMC9671963; doi:10.1038/s41467-022-34696-6)
Supplement: Supplementary file 5 — Reporting Summary [file 41467_2022_34696_MOESM5_ESM.pdf]

nature portfolio

Corresponding author(s): Sheng Ye; Shan Wu; Anna Qiao

Last updated by author(s): Jan 31, 2022

Reporting Summary

Nature Portfolio wishes to improve the reproducibility of the work that we publish. This form provides structure for consistency and transparency in reporting. For further information on Nature Portfolio policies, see our Editorial Policies and the Editorial Policy Checklist.

Statistics

For all statistical analyses, confirm that the following items are present in the figure legend, table legend, main text, or Methods section.

n/a	Confirmed	
☐	☒	The exact sample size (*n*) for each experimental group/condition, given as a discrete number and unit of measurement
☐	☒	A statement on whether measurements were taken from distinct samples or whether the same sample was measured repeatedly
☒	☐	The statistical test(s) used AND whether they are one- or two-sided<br>*Only common tests should be described solely by name; describe more complex techniques in the Methods section.*
☐	☒	A description of all covariates tested
☐	☒	A description of any assumptions or corrections, such as tests of normality and adjustment for multiple comparisons
☐	☒	A full description of the statistical parameters including central tendency (e.g. means) or other basic estimates (e.g. regression coefficient) AND variation (e.g. standard deviation) or associated estimates of uncertainty (e.g. confidence intervals)
☐	☒	For null hypothesis testing, the test statistic (e.g. *F*, *t*, *r*) with confidence intervals, effect sizes, degrees of freedom and *P* value noted<br>*Give P values as exact values whenever suitable.*
☒	☐	For Bayesian analysis, information on the choice of priors and Markov chain Monte Carlo settings
☒	☐	For hierarchical and complex designs, identification of the appropriate level for tests and full reporting of outcomes
☒	☐	Estimates of effect sizes (e.g. Cohen's *d*, Pearson's *r*), indicating how they were calculated

Our web collection on statistics for biologists contains articles on many of the points above.

Software and code

Policy information about availability of computer code

Data collection	EPU 2.9
Data analysis	The following softwares were used in Cryo--EM data processing and model building :MotionCor2 1.3.1, Gctf 1.18, RELION 3.1.1, cryoSPARC 3.1.0, Pymol and Coot. The signaling data were analyzed by Graphpad Prism 8.0. And the expression data were analyzed by Flowjo .

For manuscripts utilizing custom algorithms or software that are central to the research but not yet described in published literature, software must be made available to editors and reviewers. We strongly encourage code deposition in a community repository (e.g. GitHub). See the Nature Portfolio guidelines for submitting code & software for further information.

Data

Policy information about availability of data

All manuscripts must include a data availability statement. This statement should provide the following information, where applicable:

- Accession codes, unique identifiers, or web links for publicly available datasets
- A description of any restrictions on data availability
- For clinical datasets or third party data, please ensure that the statement adheres to our policy

Structure coordinates and structure factors have been deposited in the protein data bank for AR231453-GPR119-Gs (PDBID 7WCN, EMDB-32425) MBX-2982-GPR119-Gs (PDBID 7WCM, EMDB-32424). The underlying Fig.3, Fig.4, and Supplementary Table 3, 4 are provided as a Source data file. Other data are available from the corresponding authors upon reasonable request.

Field-specific reporting

Please select the one below that is the best fit for your research. If you are not sure, read the appropriate sections before making your selection.

- [x] Life sciences
- [ ] Behavioural & social sciences
- [ ] Ecological, evolutionary & environmental sciences

For a reference copy of the document with all sections, see nature.com/documents/nr-reporting-summary-flat.pdf

Life sciences study design

All studies must disclose on these points even when the disclosure is negative.

Sample size	3159 images for AR231453-GPR119-Gs complex and 4729 images for MBX-2982-GPR119-Gs complex were collected and analyzed. we get structures with high resolution from these data, illustrating these data are sufficient.
Data exclusions	We selected particles that are intact complex.
Replication	In AR231453/MBX-2982-induced cAMP assay, we repeated 18 times for WT. In OEA-induced cAMP assay, We repeated 6 times for WT. And other mutants were repeated 3 times. All attempts at replication were successful.
Randomization	Randomization is not relevant to this study, as protein samples are not required to be allocated into experimental groups in protein structural studies, and no animals or human research participants are involved in this study.
Blinding	yes, we used software to allocation

Reporting for specific materials, systems and methods

We require information from authors about some types of materials, experimental systems and methods used in many studies. Here, indicate whether each material, system or method listed is relevant to your study. If you are not sure if a list item applies to your research, read the appropriate section before selecting a response.

Materials & experimental systems

n/a	Involved in the study	
[ ]	[x]	Antibodies
[ ]	[x]	Eukaryotic cell lines
[x]	[ ]	Palaeontology and archaeology
[x]	[ ]	Animals and other organisms
[x]	[ ]	Human research participants
[x]	[ ]	Clinical data
[x]	[ ]	Dual use research of concern

Methods

n/a	Involved in the study	
[x]	[ ]	ChIP-seq
[ ]	[x]	Flow cytometry
[x]	[ ]	MRI-based neuroimaging

Antibodies

Antibodies used	anti-FLAG M2–fluorescein isothiocyanate antibody (Sigma-Aldrich), Cat# F4049, Antibody was diluted in by tris-buffered saline (v/v, 1:1000) and supplemented with 4% (w/w) bovine serum albumin (BSA)
Validation	The antibody has been commercially obtained and validated by the vendor. validation data are avaliable from the respective manufacturer's website, https://www.sigmaaldrich.cn/CN/zh/product/sigma/f4049

Eukaryotic cell lines

Policy information about cell lines

Cell line source(s)	HEK 293; sf9 cell ; High five cell
Authentication	cell lines were not authenticated
Mycoplasma contamination	confirm that all cell lines tested negative for mycoplasma contamination
Commonly misidentified lines (See ICLAC register)	NO

Flow Cytometry

Plots

Confirm that:

- [x] The axis labels state the marker and fluorochrome used (e.g. CD4-FITC).
- [x] The axis scales are clearly visible. Include numbers along axes only for bottom left plot of group (a 'group' is an analysis of identical markers).
- [x] All plots are contour plots with outliers or pseudocolor plots.
- [x] A numerical value for number of cells or percentage (with statistics) is provided.

Methodology

Sample preparation	we use the HEK293 cells that expressed different constructs of GPR119
Instrument	BD-FACSCalibur
Software	Flowjo
Cell population abundance	2000 cells for each test
Gating strategy	Single parameter histograms can be used to further identify distinct cell types that antibody-specific population of cells. Cells expressed GPR119 are gated according to negative cells without fluorescein isothiocyanate.

- [x] Tick this box to confirm that a figure exemplifying the gating strategy is provided in the Supplementary Information.
